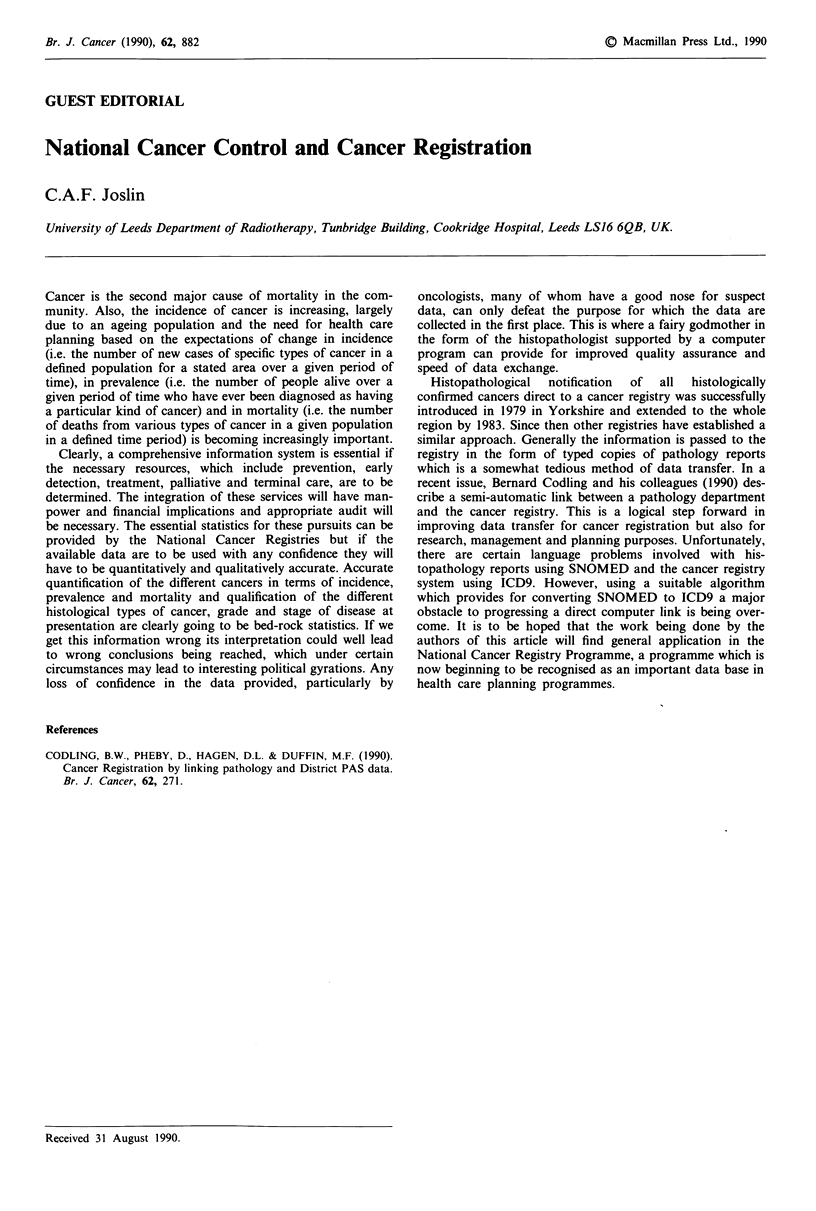# National cancer control and cancer registration.

**DOI:** 10.1038/bjc.1990.401

**Published:** 1990-12

**Authors:** C. A. Joslin


 © Macmillan Press Ltd., 1990

### GUEST EDITORIAL

### National Cancer Control and Cancer Registration

C.A.F. Joslin

*University of Leeds Department of Radiotherapy, Tunbridge Building, Cookridge Hospital, Leeds LS16 6QB, UK.*

Cancer is the second major cause of mortality in the community. Also, the incidence of cancer is increasing, largely due to an ageing population and the need for health care planning based on the expectations of change in incidence (i.e. the number of new cases of specific types of cancer in a defined population for a stated area over a given period of time), in prevalence (i.e. the number of people alive over a given period of time who have ever been diagnosed as having a particular kind of cancer) and in mortality (i.e. the number of deaths from various types of cancer in a given population in a defined time period) is becoming increasingly important.

Clearly, a comprehensive information system is essential if the necessary resources, which include prevention, early detection, treatment, palliative and terminal care, are to be determined. The integration of these services will have manpower and financial implications and appropriate audit will be necessary. The essential statistics for these pursuits can be provided by the National Cancer Registries but if the available data are to be used with any confidence they will have to be quantitatively and qualitatively accurate. Accurate quantification of the different cancers in terms of incidence, prevalence and mortality and qualification of the different histological types of cancer, grade and stage of disease at presentation are clearly going to be bed-rock statistics. If we get this information wrong its interpretation could well lead to wrong conclusions being reached, which under certain circumstances may lead to interesting political gyrations. Any loss of confidence in the data provided, particularly by oncologists, many of whom have a good nose for suspect data, can only defeat the purpose for which the data are collected in the first place. This is where a fairy godmother in the form of the histopathologist supported by a computer program can provide for improved quality assurance and speed of data exchange.

Histopathological notification of all histologically confirmed cancers direct to a cancer registry was successfully introduced in 1979 in Yorkshire and extended to the whole region by 1983. Since then other registries have established a similar approach. Generally the information is passed to the registry in the form of typed copies of pathology reports which is a somewhat tedious method of data transfer. In a recent issue, Bernard Codling and his colleagues (1990) describe a semi-automatic link between a pathology department and the cancer registry. This is a logical step forward in improving data transfer for cancer registration but also for research, management and planning purposes. Unfortunately, there are certain language problems involved with histopathology reports using SNOMED and the cancer registry system using ICD9. However, using a suitable algorithm which provides for converting SNOMED to ICD9 a major obstacle to progressing a direct computer link is being overcome. It is to be hoped that the work being done by the authors of this article will find general application in the National Cancer Registry Programme, a programme which is now beginning to be recognised as an important data base in health care planning programmes.

### References

CODLING, B.W., PHEBY, D., HAGEN, D.L. & DUFFIN, M.F. (1990). Cancer Registration by linking pathology and District PAS data. *Br. J. Cancer*, **62**, 271.

Received 31 August 1990.